# Relationships among Egg Size, Composition, and Energy: A Comparative Study of Geminate Sea Urchins

**DOI:** 10.1371/journal.pone.0041599

**Published:** 2012-07-23

**Authors:** Justin S. McAlister, Amy L. Moran

**Affiliations:** Department of Biological Sciences, Clemson University, Clemson, South Carolina, United States of America; Ecole Normale Supérieure de Lyon, France

## Abstract

Egg size is one of the fundamental parameters in the life histories of marine organisms. However, few studies have examined the relationships among egg size, composition, and energetic content in a phylogenetically controlled context. We investigated the associations among egg size, composition, and energy using a comparative system, geminate species formed by the closure of the Central American Seaway. We examined western Atlantic (WA) and eastern Pacific (EP) species in three echinoid genera, *Echinometra*, *Eucidaris,* and *Diadema*. In the genus with the largest difference in egg size between geminates (*Echinometra*), the eggs of WA species were larger, lipid rich and protein poor compared to the smaller eggs of their EP geminate. In addition, the larger WA eggs had significantly greater total egg energy and summed biochemical constituents yet significantly lower egg energy density (energy-per-unit-volume). However, the genera with smaller (*Eucidaris*) or no (*Diadema*) differences in egg size were not significantly different in summed biochemical constituents, total egg energy, or energy density. Theoretical models generally assume a strong tradeoff between egg size and fecundity that limits energetic investment and constrains life history evolution. We show that even among closely-related taxa, large eggs cannot be assumed to be scaled-up small eggs either in terms of energy or composition. Although our data comes exclusively from echinoid echinoderms, this pattern may be generalizable to other marine invertebrate taxa. Because egg composition and egg size do not necessarily evolve in lockstep, selective factors such as sperm limitation could act on egg volume without necessarily affecting maternal or larval energetics.

## Introduction

Egg size has long played an important role in the conceptual framework built around the evolution of life histories of marine organisms [1]–[8]. Among marine invertebrates, species that develop from small eggs have widely-dispersing, long-lived larvae that require planktonic feeding ( =  planktotrophic) to reach metamorphosis, whereas species that develop from large eggs are comparatively short-lived as larvae, have reduced dependence on exogenous food, or lack dispersive larvae altogether ( =  lecithotrophic) prior to metamorphosis [1], [5], [6], [9]. Large eggs are generally thought to represent greater maternal investment, and this assumption is supported by taxonomically wide-reaching studies of echinoderms in which, when comparing among species, total egg energy increases with egg biochemical constituent content [10], [11]. However, egg size can be a poor predictor of organic content across species [11], among populations [12], and among eggs produced by single individuals [13].

Large egg size is associated with numerous life history traits that are thought to reflect increases in egg energy, including reduced length of larval development [1]–[3], [5], [14]–[16], larger initial larval size [17], [18], increases in the length of the facultative feeding period [19] and of size at metamorphosis [5], [6], and faster juvenile growth with better survival [20]. Though much attention has focused on the relationship between egg size and key larval life history characters like those listed above, we understand considerably less about the associations among egg size, egg composition, and egg energy. While egg size is a comparatively simple metric to obtain, egg composition is less frequently measured because it is considerably more time- and resource- intensive. The three primary biochemical constituents of eggs of marine invertebrates are protein, lipid, and carbohydrate [10], [21], [22]; these provide the major energetic and structural elements for larval morphogenesis and development and can be measured in a variety of ways (see [23] for review). Evolutionary or plastic changes to the ratios of these constituents can drive changes in energy content independently of volume, because protein, lipid, and carbohydrate differ in energy density. For a given mass, lipid contains approximately 1.7X and 2.3X more energy than protein and carbohydrate, respectively [24]. Thus, if constituent composition changes independently of egg size, this will lead to nonlinear relationships between size and energy among taxa [25], [26]. However, little is known about the extent to which egg size and egg composition are functionally or evolutionarily linked.

Analyses of egg composition within phylogenetic frameworks are rare, but the asterinid sea stars provide one comparative example of how composition and size can be unlinked [27]. In the asterinids, the evolution of lecithotrophic larval development has generally been accompanied by both an increase in egg size and an increase in the lipid-to-protein ratio; the increase in lipid is thought to function as an energy reserve that is carried over into the post-metamorphic stage [27]. Because lipid is energy-rich compared to protein and carbohydrate, the eggs of non-planktonic feeding ( =  lecithotrophic) species are generally more energy dense than those of planktotrophs in this group. One exception was a species with unusual benthic lecithotrophic development, *Parvulastra exigua*. The eggs of this species are anomalously protein-rich and lipid-poor (and therefore have low energy density) for lecithotrophic asterinids, likely because benthic development puts a selective premium on high protein content to make embryos negatively buoyant [27]. Thus, while there was general concurrence between evolutionary shifts in egg size and composition, this relationship was also influenced by the natural history of different species.

Another example in which egg size and egg composition might not evolve in parallel comes from organisms with external fertilization. Larger eggs are known to provide better targets for sperm [7], [28], [29]; in a sperm-limiting environment, therefore, selection for increased fertilization success favors females who produce larger eggs [28], [30], [31]. If large eggs were energetically and compositionally scaled-up versions of small eggs, the energetically-driven tradeoff between the production of larger eggs and decreased fecundity would reduce the fitness benefits of increased fertilization success [7], [10], [11]. If, however, larger eggs had lower energy densities (energy per unit volume) than smaller eggs, this would alleviate constraints on the evolution of egg size imposed by tradeoffs between fertilization success and high fecundity [7], [10], [32], [33]. Despite considerable interest in these ideas and the fundamental ecological and evolutionary importance of determining the links between egg size, egg composition, and egg energy, few empirical studies have examined the relationship between these traits in a comparative evolutionary framework.

We addressed the following question: are there predictable relationships between egg size, egg composition, and egg energy in planktotrophic species? We investigated this question in the context of geminate sea urchins, which are sister taxa separated by the Central American Isthmus (CAI) that rose approximately 2–4 million years ago [34] and divided a once continuous marine environment into the tropical western Atlantic (WA) and eastern Pacific (EP) oceans [35]. Geminate species pairs occur in multiple phyla [35], and while divergence times often predate the rise of the Isthmus [36], [37], geminates have been evolving in isolation for at least 3 million years since the final closure of the Central American Seaway (CAS) [38]. In several phyla of invertebrates, most WA species have larger eggs than their EP geminates [39]–[41]. This pattern has been attributed to increased maternal investment per egg in the WA to compensate for a comparatively low-productivity, low-food environment [39], and/or reduced maternal investment per egg in the EP, where productivity is higher, to enhance female fecundity [41]. Though eggs are generally larger in the WA when geminates are compared, there is no characteristic ‘optimal’ egg size that all taxa converge on within either ocean; instead, the egg size of a particular taxon reflects the influence of both environmental changes and lineage-dependent phylogenetic constraints [39].

We measured egg size and biochemical composition and calculated the egg energy and energy density in seven geminate species from three genera: *Diadema antillarum* (WA) and *D. mexicanum* (EP), *Eucidaris tribuloides* (WA) and *Eu. thouarsii* (EP), and the *Echinometra* triplex containing the sympatric sister taxa *Ec. lucunter* and *Ec. viridis* (WA), and *Ec. vanbrunti* (EP). In all three genera, molecular evidence supports a sister-species relationship between geminates, with genetic divergence having likely occurred between WA and EP species around the time of final closure of the CAS ∼3.2 million years ago [42]–[44]; in the *Echinometra* triplex, the age of the split between the two WA taxa is approximately half that of the EP-WA split (1.27–1.62 mya), suggesting the WA pair formed via a post-Isthmian speciation event in the Caribbean [43]. All seven species possess a long-lived plankton-feeding larval stage. In one study [39], eggs of the WA *Echinometra* were approximately 2X greater in volume (a significant difference) than their EP geminate; in the *Eucidaris* pair the WA species also had significantly larger eggs, though this difference was comparatively smaller (∼11%). *Diadema* was the only echinoderm genus in Lessios’ [39] study that showed the reverse pattern, with slightly but significantly larger (∼7%) eggs in the EP.

## Materials and Methods

Adult sea urchins were collected from coastal waters near the Naos Island Laboratories (EP) and Galeta Marine Laboratory (WA) of the Smithsonian Tropical Research Institute (STRI) in the Republic of Panama. All necessary permits were obtained for the described field studies. Collections made in 2005 and 2008 at Isla Taboguilla (EP) and Punta Galeta (WA) were conducted under an annual resolution signed by the Director General de Recursos Marinos y Costeros de la Autoridad Marítima de Panamá (AMP) authorizing collection of marine organisms by staff and visiting scientists associated with STRI. Collections made at the same locations in 2010 were conducted under collection permit SC/A-14-10 issued by the Autoridad Nacional del Ambiente (ANAM). Gametes were obtained by injecting adult urchins with 0.5 M KCl. Single axis diameters of 25 eggs from each female (5–7 females per species) were measured using an ocular micrometer (for samples collected in 2005 and 2008) or from digital images of eggs (for samples collected in 2010) using ImageJ software (National Institutes of Health). For measurement, eggs were suspended in filtered seawater on glass slides under cover slips on clay feet to prevent flattening of the eggs. Egg volumes (nl) were calculated as for a sphere using egg diameters.

We assayed the eggs of each female for total protein, lipid, and carbohydrate, which are the primary energetic reserves contained in the eggs of marine invertebrates [10], [21], [22]. Nine replicate samples of known egg number (1500–5000/sample) were collected from each of the five to seven females of each species. Samples were frozen at −80°C and transported to Clemson University for biochemical analysis, and three samples were used as replicates for each assay. Protein was assayed using a micro-modification of the Lowry Protein Assay ([45]; available commercially as the Micro BCA Protein Assay Kit from Pierce). Total lipid was estimated by the sulfuric acid charring technique of Marsh and Weinstein [46] as modified by Holland and Gabbott [21]. Carbohydrate content was assayed using a potassium ferricyanide sodium carbonate/cyanide reducing reaction [21], [47]. These assays have a long history in the literature (e.g. [48]–[52]; see [10] and [23] for review), so our sampling methodology and data can be directly compared with much of the previous literature on invertebrate eggs.

We tested for differences in egg volume, biochemical constituents contents, and total energy content between species within each genus (with the calculated grand mean of samples from each mother representing an independent datum) using one-way analyses of variance (PROC MIXED: SAS Institute, Cary, NC). Because our initial expectation was that larger eggs would contain more energy and more of each constituent, we used one-tailed ANOVAs for these comparisons. For *Diadema*, we conducted two-tailed ANOVAs for biochemical constituents and energy. Because we found no significant difference in egg size for this genus, we had no *a priori* expectations about biochemical or energy content. We also tested for differences in density (ng nl^−1^) of each biochemical constituent, proportional contribution of each constituent towards total biochemical composition, total energy density, lipid-to-protein ratio, and the proportional contribution towards total energy from lipid using one-way ANOVAs (PROC MIXED: SAS Institute, Cary, NC). Because we had no *a priori* expectation for directionality in these comparisons, we used two-tailed ANOVAs. In all tests, degrees of freedom were calculated using the DDFM  =  SATTERTH (Satterthwaite approximation) option. Data normality was tested using Shapiro-Wilk (PROC UNIVARIATE: SAS Institute, Cary, NC).

## Results

**Figure 1:**
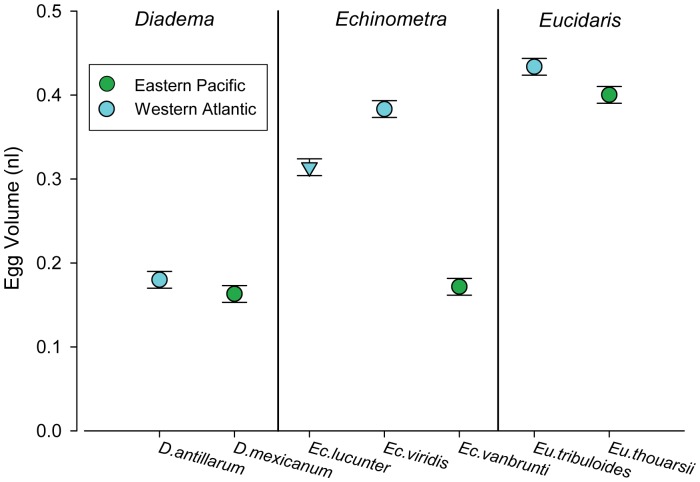
Egg volume. Average (1± SE) egg volume for Eastern Pacific (green) and Western Atlantic (blue) species of sea urchins in the genera *Diadema*, *Echinometra*, and *Eucidaris*.

In the *Echinometra* triplex and in *Eucidaris*, eggs of the WA species were significantly larger than eggs of their EP geminates (Fig. 1 and Tables 1,2). There were no significant egg size differences between geminate *Diadema*. The relationships between egg size, egg composition, and egg energy density varied among the three sets of geminates. We present results for each genus in turn below.

**Table 1:** Average (±1 SE) egg volumes (nl), individual biochemical constituent contents (ng egg^−1^) and densities (ng nl^−1^), lipid:protein ratios, total biochemical constituent content (ng egg^−1^), total energy (mJ egg^−1^), and energy densities (mJ nl^−1^).

	Species						
	*Ec. lucunter* (WA)	*Ec. viridis* (WA)	*Ec. vanbrunti* (EP)	*Eu. tribuloides* (WA)	*Eu. thouarsii* (EP)	*D. antillarum* (WA)	*D. mexicanum* (EP)
Egg Volume (nl)	0.31 (0.01)	0.38 (0.01)	0.17 (0.01)	0.43 (0.01)	0.40 (0.01)	0.18 (0.01)	0.16 (0.01)
Total Lipid (ng egg^−1^)	17.1 (1.3)	15.4 (1.3)	9.4 (1.3)	19.0 (1.3)	18.1 (1.1)	9.8 (1.3)	8.1 (1.3)
Lipid density (ng nl^−1^)	54.3 (6.0)	40.7 (6.0)	56.1 (6.0)	43.6 (2.9)	45.3 (2.5)	53.8 (6.0)	49.6 (6.0)
% Lipid	30.7 (1.1)	27.9 (1.1)	21.5 (1.1)	30.5 (2.1)	27.6 (1.8)	28.1 (2.6)	25.5 (2.6)
Total Protein (ng egg^−1^)	35.3 (2.1)	35.8 (2.1)	29.9 (2.1)	38.4 (2.8)	43.5 (2.4)	19.9 (1.4)	18.5 (1.4)
Protein density (ng nl^−1^)	111.6 (9.2)	93.9 (9.2)	175.9 (9.2)	89.2 (6.6)	108.7 (5.6)	111.4 (7.1)	112.9 (7.1)
% Protein	63.3 (1.5)	65.2 (1.5)	69.4 (1.5)	61.1 (2.2)	66.2 (1.8)	57.4 (1.7)	59.2 (1.7)
Total Carbohydrate (ng egg^−1^)	3.3 (0.6)	3.7 (0.6)	4.1 (0.6)	5.3 (0.4)	4.1 (0.3)	4.9 (0.8)	4.7 (0.8)
Carbohydrate density (ng nl^−1^)	10.8 (3.6)	10.1 (3.6)	24.2 (3.6)	12.2 (1.2)	10.5 (1.0)	28.7 (5.2)	29.1 (5.2)
% Carbohydrate	5.98 (0.9)	6.88 (0.9)	9.05 (0.9)	8.4 (0.5)	6.2 (0.4)	14.5 (2.6)	15.3 (2.6)
Summed Constituents (ng egg^−1^)	55.7 (3.4)	54.9 (3.4)	43.4 (3.4)	62.7 (3.2)	65.6 (2.7)	34.6 (2.1)	31.3 (2.1)
Lipid:Protein	0.49 (<.1)	0.43 (<.1)	0.31 (<.1)	0.52 (0.1)	0.42 (<.1)	0.49 (0.1)	0.44 (0.1)
Total Energy (mJ egg^−1^)	1.6 (0.1)	1.5 (0.1)	1.2 (0.1)	1.8 (0.1)	1.8 (0.1)	1.0 (0.1)	0.9 (0.1)
Energy density (mJ nl^−1^)	5.0 (0.5)	4.0 (0.5)	6.9 (0.5)	4.1 (0.2)	4.6 (0.2)	5.3 (0.3)	5.2 (0.3)
% Energy from Lipid	42.7 (1.5)	39.5 (1.5)	31.8 (1.5)	42.5 (2.5)	39.1 (2.1)	40.2 (2.9)	37.2 (2.9)

**Table 2:** Formal statistical results from one-way ANOVAs using Restricted Mean Likelihood (*indicates one-tailed tests used for *Echinometra* and *Eucidaris.* All tests used for *Diadema* were two-tailed.).

	Comparison				
	*Ec. lucunter* (WA) *–* *Ec. vanbrunti* (EP)	*Ec. viridis* (WA) *–* *E. vanbrunti* (EP)	*Ec. lucunter* (WA) *– Ec. viridis* (WA)	*Eu. tribuloides* (WA) *–* *Eu. thouarsii* (EP)	*D. antillarum* (WA) *– D. mexicanum* (EP)
Degrees of freedom for all tests	n df = 1, d df = 10	n df = 1, d df = 10	n df = 1, d df = 10	n df = 1, d df = 10	n df = 1, d df = 8
Egg Volume (nl)*	t = 9.32, **p<0.01**	t = 14.95, **p<0.01**	t = 3.52, **p<0.01**	t = 1.94, **p** = **0.04**	F = 1.42, p = 0.27
Total Lipid (ng egg^−1^)*	t = 4.22, **p<0.01**	t = 3.42, **p<0.01**	t = −0.86, p = 0.80	t = 0.51, p = 0.31	F = 0.90, p = 0.37
Lipid density (ng nl^−1^)	F = 0.05, p = 0.84	F = 3.29, p = 0.09	F = 7.26, **p** = **0.02**	F = 0.18, p = 0.68	F = 0.25, p = 0.63
% Lipid	F = 32.60, **p<0.01**	F = 17.43, **p<0.01**	F = 2.01, p = 0.19	F = 1.11, p = 0.32	F = 0.49, p = 0.50
Total Protein (ng egg^−1^)*	t = 1.72, **p** = **0.05**	t = 2.19, **p** = **0.03**	t = 0.17, p = 0.44	t = −1.37, p = 0.90	F = 0.50, p = 0.50
Protein density (ng nl^−1^)	F = 16.74, **p<0.01**	F = 28.75, **p<0.01**	F = 11.42, **p<0.01**	F = 5.07, **p** = **0.05**	F = 0.02, p = 0.88
% Protein	F = 8.28, **p** = **0.02**	F = 3.60, p = 0.09	F = 0.87, p = 0.37	F = 3.16, p = 0.11	F = 0.53, p = 0.49
Total Carbohydrate (ng egg^−1^)*	t = −0.89, p = 0.81	t = −0.36, p = 0.64	t = 0.80, p = 0.22	t = 2.31, **p** = **0.02**	F = 0.03, p = 0.86
Carbohydrate density (ng nl^−1^)	F = 6.80, **p** = **0.03**	F = 7.63, **p** = **0.02**	F = 0.13, p = 0.73	F = 1.23, p = 0.29	F = 0.00, p = 0.96
% Carbohydrate	F = 5.26, **p** = **0.04**	F = 2.30, p = 0.16	F = 0.66, p = 0.44	F = 10.36, **p<0.01**	F = 0.05, p = 0.83
Summed constituents (ng egg^−1^)*	t = 2.38, **p** = **0.02**	t = 2.45, **p** = **0.02**	t = −0.17, p = 0.56	t = −0.70, p = 0.75	F = 1.19, p = 0.31
Lipid:Protein	F = 24.40, **p<0.01**	F = 11.74, **p** = **0.01**	F = 1.60, p = 0.24	F = 2.11, p = 0.18	F = 0.55, p = 0.48
Total Energy (mJ egg^−1^)*	t = 2.86, **p<0.01**	t = 2.77, **p<0.01**	t = −0.36, p = 0.64	t = −0.59, p = 0.71	F = 1.13, p = 0.30
Energy density (mJ nl^−1^)	F = 5.84, **p** = **0.04**	F = 13.97, **p<0.01**	F = 12.00, **p<0.01**	F = 3.51, p = 0.09	F = 0.07, p = 0.80
% Energy from Lipid	F = 31.89, **p<0.01**	F = 16.74, **p<0.01**	F = 1.91, p = 0.20	F = 1.09, p = 0.32	F = 0.54, p = 0.48

Abbreviations are: degrees of freedom (df), numerator (n), denominator (d), t-statistic (t), F-statistic (F), p-value (p). Bold text indicates p-values ≤ α level 0.05.

### Echinometra

**Figure 2:**
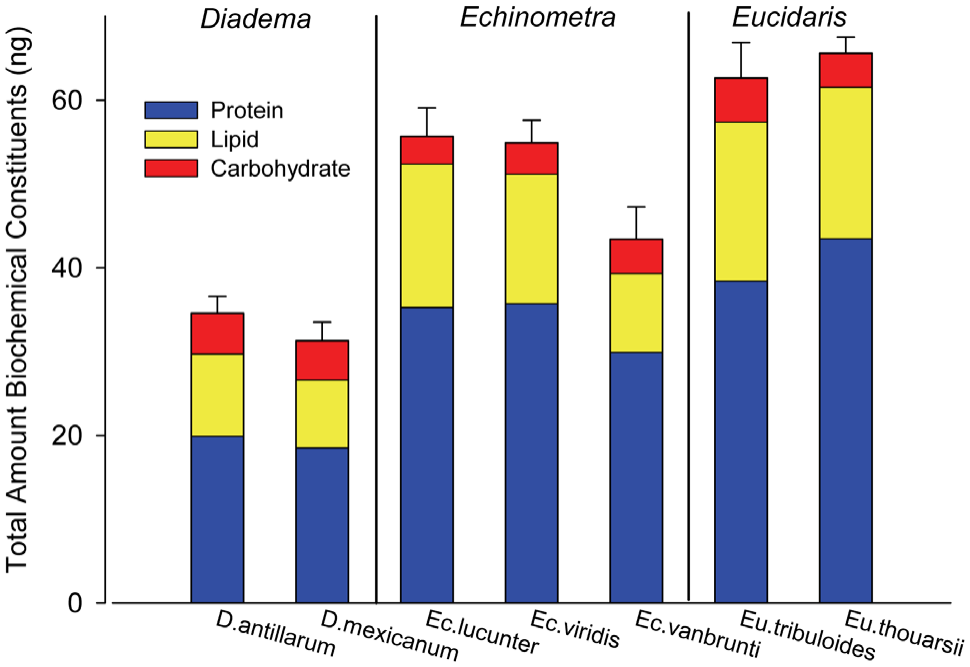
Mean biochemical constituent content of eggs. Average (±1 SE) summed total amounts (ng) of biochemical constituents per egg per species. Average protein (blue), lipid (yellow), and carbohydrate (red) content per egg per species are indicated.

In *Echinometra*, both WA species had significantly larger eggs (mean ± SE) than *Ec. vanbrunti* (EP): *Ec. lucunter* eggs (0.31±0.01 nl) and *Ec. viridis* eggs (0.38±0.01 nl) were 1.8x and 2.2x larger than *Ec. vanbrunti* eggs (0.17±0.01 nl), respectively (ANOVA, t = 9.32, df = 10, p<0.01 and t = 14.95, df = 10, p<0.01 for respective WA to EP comparisons, Fig.1 and Tables 1,2). Within the WA, although the eggs of *Ec. viridis* were significantly larger (by 1.2x; ANOVA, t = 3.52, df = 10, p<0.01) than *Ec. lucunter* eggs (Fig.1, Tables 1,2), we found no significant differences between these species’ eggs in individual or summed constituent content, total energy, and/or energy density (Fig. 2, Tables 1,2). We did find however, that the larger eggs of *Ec. viridis* were significantly less lipid and protein dense than the smaller eggs of sympatric *Ec. lucunter* (Tables 1,2). The eggs of both WA species of *Echinometra* contained significantly more lipid and protein than their EP counterpart (Fig. 2; Tables 1,2). Carbohydrate content was not significantly different among the three species. Proportionally, the two WA *Echinometra* species’ eggs were significantly more lipid-rich and protein-poor than *Ec. vanbrunti* (EP) (Tables 1,2). *Ec. lucunter* (WA) eggs were significantly less carbohydrate-rich than *Ec. vanbrunti* (EP); there was no significant difference in the proportions of carbohydrate in eggs of *Ec. viridis* (WA) and *Ec. vanbrunti* (EP) (Tables 1,2).

**Figure 3:**
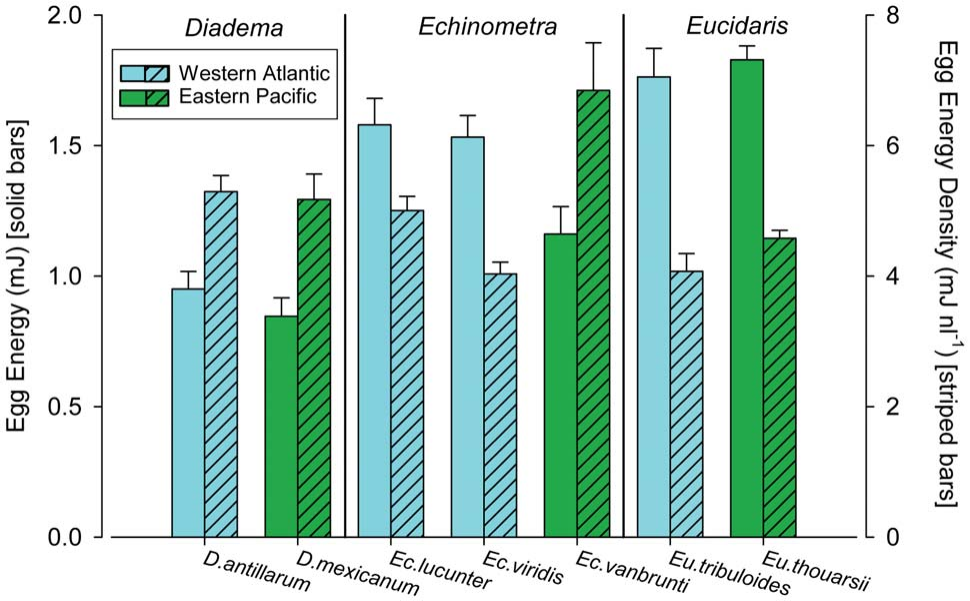
Average energy and energy density of eggs. Left vertical axis and non-patterned bars: Average (±1 SE) total energy content (mJ) per egg per species. Enthalpic energy equivalents of 39.5 µJ/ng (lipid), 24 µJ/ng (protein), and 17.5 µJ/ng (carbohydrate) were used to calculate energy content. Right vertical axis and patterned bars: Average (±1 SE) energy density (mJ nl^−1^) per egg per species. Western Atlantic species (blue) and Eastern Pacific species (green) are indicated.

When we summed the oxyenthalpic energy equivalents of each biochemical constituent, protein (24 kJ/g), carbohydrate (17.5 kJ/g), and lipid (39.5 kJ/g) (24), we found that the eggs of both WA *Echinometra* contained significantly more total energy (Fig. 3 and Tables 1, 2) than *Ec. vanbrunti* (EP) eggs. Egg energy densities (calculated as egg energy content divided by egg volume (mJ nl^−1^)) of the two WA *Echinometra* species were both significantly lower than *Ec. vanbrunti* (EP) (Fig. 3, Tables 1,2 ); eggs of *Ec. lucunter* and *Ec. viridis* had energy densities that were 0.72x and 0.58x the energy density of *Ec. vanbrunti* (Fig. 3; Tables 1, 2). The WA species’ eggs were not significantly different from each other in total energy but the smaller eggs of *Ec. lucunter* were significantly more energy dense than the larger eggs of *Ec. viridis* (Fig. 3; Tables 1,2).

### Eucidaris

Egg volume of *Eu. tribuloides* (WA: 0.43±0.01 nl) was significantly larger than *Eu. thouarsii* (EP: 0.40±0.01 nl) (ANOVA, t = 1.94, df = 10, p = 0.04; Fig. 1; Tables 1,2). There were no significant differences in lipid and protein content between species (Tables 1,2). The eggs of *Eu. tribuloides* contained significantly more carbohydrate than eggs of *Eu. thouarsii,* both quantitatively and as a proportion of total summed biochemical constituents (Fig. 2 and Tables 1,2). There were no significant differences between the two species in total egg energy (mJ) or energy density (mJ nl^−1^) (Fig. 3; Tables 1,2).

### Diadema

The eggs of *D. antillarum* (WA: 0.18±0.01 nl) and *D. mexicanum* (EP: 0.16±0.01 nl) were not significantly different in volume (ANOVA, F = 1.42, df = 8, p = 0.27; Fig. 1; Tables 1,2). Similarly, differences in both the absolute and proportional contents of biochemical constituents were small between *Diadema* geminates and none were significant (Fig. 2; Tables 1,2). We found no significant differences in total energy or energy density between the eggs of *D. antillarum* (WA) and *D. mexicanum* (EP) (Figs. 2,3; Tables 1,2). We present results of two-tailed tests for all comparisons for this genus (Table 2), but obtained similarly non-significant results using one-tailed tests for biochemical constituent and total energy comparisons.

## Discussion

To date, most biochemical analyses of egg composition (as it relates to egg size) have focused on lecithotrophic species or comparisons between planktotrophs and lecithotrophs [26], [53]–[56]. By comparing the egg energy and biochemical profiles of eggs of closely-related planktotrophs in a phylogenetically-controlled context, our data provide what is to our knowledge the first examination of how these parameters are related to each other in small eggs and over comparatively short, defined evolutionary timescales.

### Biomass, Energy, and Size

One of the primary assumptions of life history theory is that larger eggs contain more biomass and more energy than small eggs [10], [11]. We found this pattern to be broadly true across the seven species we examined; overall, larger eggs contained more material and more energy than smaller eggs (Figs. 1,2,3), and egg volume explained 88% of the variance in egg energy across all 7 species (linear regression of (ln) energy vs. (ln) volume, y = 0.60x+1.09, r^2^ = 0.88, p<0.01). In *Echinometra*, which had significant differences in egg size among geminates, the species with larger eggs (WA) contained significantly more biomass and energy than their EP counterpart (Figs. 2,3, Tables 1,2). In *Eucidaris,* though eggs of the WA species were significantly larger, we found no significant differences in lipid, protein, summed constituent, or energy contents, or in energy density (Figs. 2,3, Tables 1,2). In *Diadema*, egg sizes were not significantly different between geminates (Fig. 1, Table 2) and neither were any indices of composition or energy. Though our ability to detect small differences was limited by sample size (which was constrained by the limits of field season and availability of ripe animals), our data suggest that as in egg size, differences in egg composition or egg energy between geminates of *Eucidaris* and *Diadema*, if present, are subtle (Figs. 2,3, Table 2).

### Biochemical Composition

Total egg energy is the currency most often considered by life history models [2]–[6], but from the perspective of a developing embryo, the partitioning of that energy among different biochemical constituents is also important. In lecithotrophic echinoderms, lipid can make up>60% of egg material [26]: lipid fuels larval development [25], [26], [54], [56], post-metamorphic development [53], and in some cases enhances fertilization and dispersal through its effects on buoyancy [26], [57], [58]. Evolutionarily, as egg size increases, the proportional amount of lipid in eggs tends to increase [10], [11]; statistically, however, this pattern may be driven primarily by measurements taken from the very large and lipid-rich eggs of many lecithotrophic echinoderms, and less is known about planktotrophic species.

Our data show that in the tropical American *Echinometra* in this study, all of which are planktotrophs, larger eggs contain proportionally more lipid. Because lipids fuel development to the end of the facultative feeding period, eggs that contain more lipid likely produce larvae that can develop further along their trajectory before needing to feed [27], [59], [60]. This is consistent with our observations of larval development in *Echinometra*, in which starved larvae of the WA species develop to later stages than their EP geminates before developmental arrest (McAlister & Moran, in prep.). The larger eggs of the two WA species of *Echinometra* also contained more protein than their EP counterpart. Protein contains only ∼60% of the energy in lipid per unit mass [16]. Egg protein can serve as an energy source [10], [61], [62], but it largely has a structural role in early development because lipid is the primary source of energy for prefeeding larvae [10], [26], [57], [63]–[67]. Our data for *Echinometra* are consistent with a structural role of egg protein for building larval bodies and arms because the WA species, which had significantly more protein in their eggs, produced bigger larvae with longer arms at the pre-feeding stage (Fig. 2, Table 1, for protein; [68] for arm length data).

We also measured carbohydrate, which typically comprises <5% of echinoderm egg biomass [10] and so is often not measured in biochemical studies of eggs and larvae [23]. In our samples, carbohydrate made up between ∼6 and 15% of egg biomass (Fig. 2, Table 1). The three species with the smallest eggs (two *Diadema* spp. and *Echinometra vanbrunti*) had the highest proportion of carbohydrate, which is consistent with a broader pattern among echinoderms of smaller eggs containing proportionally more of this constituent [10]. The jelly coat that surrounds and protects echinoderm eggs prior to hatching is made largely of carbohydrate in the form of polysaccharides [69], and the jelly coat contains between 3 and 18% of total egg energy [70]. Carbohydrate is also the main component of the hyaline layer that surrounds the embryo during development [71], [72]. While we did not directly measure jelly coats or the hyaline layer, all else being equal, surface-area-to-volume scaling relationships would dictate that smaller eggs contain higher proportions of carbohydrate. Because the jelly coat does not provide nutrition to larvae [70], [73], differences in quantity of carbohydrate between eggs probably largely reflects scaling, selection for fertilization success [7], [8], [28], [29], [31]–[33] or mechanical protection of the egg and embryo [71], [74] rather than an energetic or structural function for larvae.

### Egg Size, Egg Energy, and Energy Density

When we compared total egg energy and energy density among the *Echinometra* spp., it was evident that egg size was not a good indicator of either among these three sister taxa. Total egg energy, calculated from the sum of the energy available from protein, lipid, and carbohydrate, was significantly greater in the larger eggs of WA species (there was no significant difference in total energy between the two WA species (Fig. 3, Tables 1,2)). However, energy density, which we calculated as total energy divided by volume, was significantly *lower* in the eggs of the two WA species compared to their EP geminate (Fig. 3, Tables 1,2). Energy density of eggs is determined by both composition, i.e. the proportions of high- and low-energy constituents (lipid vs. protein, for example), and by hydration [33]. In *Echinometra,* hydration is likely an important determinant of energy density because the WA species, which have low energy densities compared to their EP geminate, contain proportionally *more* energy-rich lipid (Table 1). In addition, though the larger eggs of *Ec. viridis* were not significantly different in composition from those of *Ec. lucunter* (Fig. 2, Tables 1,2), they were significantly less lipid, protein, and energy dense, suggesting that the eggs of *Ec. viridis* may be hydrated to a greater degree than those of its sympatric sister species. Thus, egg size in this genus likely reflects other selective agents that go beyond the energetic and structural needs of developing larvae.

Why would energy density of eggs vary between species? Most life history models assume that evolutionary changes in egg size are related to the energetic and structural demands of larval development in different environments [2]–[6], but egg size also has important implications for fertilization success. In a sperm-limited environment, larger eggs are better targets for sperm [8]. While the increase in gamete production from higher fertilization rates could be offset by a reduction in fecundity due to the increased cost of making larger individual eggs [7], through hydration females could make larger eggs and increase their fertilization success while avoiding the full reduction in fecundity they would incur if large eggs were scaled-up small eggs. While we have no *a priori* reason to think that sperm limitation underlies the egg size differences between taxa, the lower energy density of the eggs of the two WA *Echinometra* species compared to *Ec. vanbrunti* (EP), coupled with lower energy density of eggs of *Ec. viridis* compared to *Ec. lucunter* within the WA, suggests that egg size may be responding to selection on target size in conjunction with the requirements of larval and embryonic energetics.

### The Productivity Hypothesis

In WA species, larger eggs are thought to have evolved in response to selection to offset the decrease in productivity - the availability of phytoplankton as larval food - in the WA that occurred after the rise of the Central American Isthmus [39], [75]–[77]. Our data for *Echinometra* are consistent with this idea, because eggs of the WA species have both more lipid and higher lipid:protein ratios than their EP geminate. This suggests that in the food-poor WA, *Echinometra* mothers supply larvae with comparatively large energetic ‘gas tanks’ relative to materials for larval construction, thus potentially reducing the dependence of larvae on exogenous food. However, these larger and more lipid-rich eggs are also less energy-dense, so other selective factors (such as fertilization success, for example, or buoyancy) are likely in play.

In a food-poor environment, mothers might also be expected to increase the amount of protein in eggs to construct larger feeding structures. Echinoid species with longer larval arms have longer ciliated bands and hence greater food-capturing capacities [78]. Of the geminates we examined, only *Echinometra* had significantly higher amounts of egg protein (along with longer arms) in the WA than in the EP. We found no significant differences in protein between WA and EP species of *Diadema*, although WA *D. antillarum* has been shown to produce longer arms than EP *D. mexicanum* [68]. The genus *Eucidaris* showed no significant differences in the amount of egg protein, but had significantly higher protein density and proportionally longer larval arms [68] in the EP species. This pattern, which is the opposite of that seen in *Echinometra*, is probably not due to differences in environment or spawning seasonality among genera, because all EP and WA species occurred sympatrically and have overlapping reproductive seasons. *Eucidaris* are not closely related to either *Echinometra* or *Diadema* [44] and their larvae are both morphologically [6] and physiologically (McAlister & Moran, in prep.) distinct, however, so even within the same environment they may experience different selective regimes for feeding- and predation-related traits such as arm length.

Lessios [39] pointed out that while there were multiple parallel shifts in egg sizes of echinoderms in the three million years following the closure of the CAI, different taxa did not converge on an ‘optimal’ egg size in each ocean and such shifts occurred “within the constraints imposed by phylogeny…and these constraints seem fairly stringent.” Thus, the productivity hypothesis, while it provides a context for understanding the repeated shifts in egg size between members of geminate pairs, cannot explain or predict egg size outside of this controlled phylogenetic context. Our data suggest that the same is true for egg biochemistry and energetics. *Echinometra*, *Eucidaris*, and *Diadema* occur sympatrically and have overlapping spawning periods within oceans [79]–[81], yet have not converged on a single egg size, or biochemical or energetic profile, in either ocean. In *Echinometra*, we found that WA eggs were up to ∼2x larger than eggs of their EP geminate, and contained more lipid, protein, and total energy, and had lower energy densities. In *Eucidaris*, we detected a significant difference between WA and EP species in egg size, but no significant differences in the amounts of lipid, protein, total energy, and/or energy density; the only significant differences were that the WA *Eucidaris* had less carbohydrate and higher protein density. In *Diadema*, there has been little measureable change between oceans; we did not find significant differences in egg size, egg composition, egg energy, or energy density. Although we did not sample across a wide geographic region and so may have missed inter-population variation in egg size or content [51], our measures of egg size were very close to those of Lessios [39] for individuals he collected from different sites and in different years. Thus, despite having shared common oceanic environments for the past ∼3.2 million years, the WA and EP members of these three genera have likely responded in markedly different ways with respect to the evolution of the size, composition, and energy content of eggs.

To test this idea, we used a two-way ANOVA (PROC MIXED, SAS Institute, Cary, NC) to identify significant effects of ocean, genus, and, most importantly, an interaction between ocean and genus, on egg energy. This analysis found significant differences due to ocean (p = .0396, F_1,34_ = 4.58), genus (p<.0001, F_2,34_ = 52.50), and the interaction of ocean-by-genus (p = .0181, F_2,34_ = 4.53). The ocean-by-genus effect was driven by the large difference between WA and EP *Echinometra* (multiple comparisons of differences of least-square means, p = .0005, t = 3.88). The significant ocean-by-genus interaction supports our hypothesis that genera have responded in lineage-specific ways to the differences in productivity (or other factors) between the WA and EP.

Our data suggest that as is the case for some lecithotrophs [26], for planktotrophic species, larval energetics and larval feeding environment, while clearly important [1]–[3], [5], [6], [9], [14]–[19], are not the only factors influencing the evolution of egg size. Phylogenetic history also appears to play a key role in constraining the evolution of both egg size [39] and egg composition; likewise, fertilization environment [7], [8] and other, non-energetic factors likely play a role. Thus, larval feeding environment should be only one of several factors considered when examining spatial and temporal patterns of egg and offspring size variation (e.g. [1], [39], [82]–[84]). Furthermore, as has been frequently pointed out [12], [13], egg size cannot safely be used as a stand-in for maternal investment, even within closely-related species; the relationships between egg size, egg composition, and egg energy in planktotrophic species are not always predictable. Additional detailed studies on the biochemistry and mechanisms of oogenesis from closely related species can provide a greater understanding of the evolutionary, physiological, and ecological links between these two important life history characters.
